# Myeloid-derived suppressor cells contribute to A2B adenosine receptor-induced VEGF production and angiogenesis in a mouse melanoma model

**DOI:** 10.18632/oncotarget.4393

**Published:** 2015-06-25

**Authors:** Claudia Sorrentino, Lucio Miele, Amalia Porta, Aldo Pinto, Silvana Morello

**Affiliations:** ^1^ Department of Pharmacy, University of Salerno, Fisciano SA, Italy; ^2^ Department of Genetics, School of Medicine, LSU Health Sciences Center, New Orleans, Louisiana, USA

**Keywords:** A2B adenosine receptor, myeloid-derived suppressor cells, tumor angiogenesis, melanoma

## Abstract

Vascular endothelial growth factor (VEGF) is an angiogenic factor critically involved in tumor progression. Adenosine A2B receptor plays a pivotal role in promoting tumor growth. The aim of this study was to investigate the role of myeloid-derived suppressor cells (MDSCs) in the pro-angiogenic effects of A2B and to determine whether A2B blockade could enhance the effectiveness of anti-VEGF treatment. Mice treated with Bay60-6583, a selective A2B receptor agonist, showed enhanced tumor VEGF-A expression and vessel density. This effect was associated with accelerated tumor growth, which could be reversed with anti-VEGF treatment. Bay60-6583 increased the accumulation of tumor CD11b+Gr1+ cells. Depletion of MDSCs in mice significantly reduced A2B-induced VEGF production. However, A2B receptor stimulation did not directly regulate VEGF expression in isolated tumor myeloid cells. Mechanistically, Bay60-6583-treated melanoma tissues showed increased STAT3 activation. Inhibition of STAT3 significantly decreased the pro-tumor activity of Bay60-6583 and reduced tumor VEGF expression.

Pharmacological blockade of A2B receptor with PSB1115 significantly reduced tumor growth by inhibiting tumor angiogenesis and increasing T cells numbers within the tumor microenvironment. These effects are, at least in part, dependent on impaired tumor accumulation of Gr1+ cells upon A2B receptor blockade. PSB1115 increased the effectiveness of anti-VEGF treatment.

## INTRODUCTION

Adenosine is an ATP-derived nucleoside, whose effects are mediated by four G-coupled receptors: cAMP-elevating A2A and A2B receptors, and A1 and A3 receptors, which reduce the levels of intracellular cAMP [1]. Under hypoxic and inflammatory conditions, damaged or metabolically stressed cells mediate an increase in extracellular hydrolysis of ATP into adenosine, protecting tissue from excessive damage [2, 3]. In cancer lesions, adenosine, produced by tumor cells and/or by tumor-infiltrating immune cells, accumulates in the microenvironment [4]. Stromal accumulation of adenosine induces strong immune suppression, promoting tumor immune escape via A2 adenosine receptors (A2A and A2B) signaling [3, 5, 6]. Genetic deletion of A2A receptor induces a strong anti-tumor T cell response, rejection of established tumors and prolonged survival in tumor-bearing hosts [7, 8]. Therefore blockade of A2A receptor has proved to be effective in preventing both the inhibition of T effector cells and the induction of T-regulatory (T-reg) cells [6].

Emerging evidence suggests that the A2B receptor is also critically involved in adenosine-induced tumor growth. Although A2B is a low-affinity adenosine receptor, its activation occurs in hypoxic tissues, including cancer, where micromolar concentrations of adenosine are achieved [6, 9]. Therefore, the A2B receptor may have a critical role in mediating adenosine effects under pathological conditions [9]. The A2B receptor participates in the pro-angiogenic effects of adenosine. Stimulation of A2B receptor promotes the release of VEGF from human endothelial cells [10, 11]. Other studies also suggest that adenosine induces VEGF release in cancer cell lines via the A2B receptor [12]. Together with endothelial cells and cancer cells, host immune cells also participate in the pro-angiogenic effects mediated by A2B receptor [13]. Novitskiy et al. [14] showed that dendritic cells, differentiated through the A2B receptor, release high levels of angiogenic factors, including VEGF and interleukin (IL)-8, and promote tumor growth when injected into mice bearing Lewis lung carcinomas. Later, the same authors demonstrated that A2B receptor-deficient mice show low levels of tumor-associated myeloid-derived suppressor cells (MDSCs) [13, 15].

MDSCs, together with T-reg cells, play a critical role in inducing immune suppression in tumor hosts [16]. MDSCs are potent suppressor of T cell-mediated responses in the tumor microenvironment, and promote tumor progression and invasion [17]. In addition, tumor-infiltrating MDSCs produce pro-angiogenic factors, such as VEGF, in a STAT3-dependent manner and stimulate tumor angiogenesis [18–20]. In turn, pro-angiogenic factors can further enhance MDSCs accumulation within tumors [18, 20, 21], creating a vicious circle. We recently demonstrated that pharmacological blockade of A2B receptor significantly reduces tumor accumulation of MDSCs and decreases the levels of inflammatory mediators, such as IL-10 and monocyte chemoattractant protein (MCP)-1 (also known as C-C motif ligand-2, CCL-2) [22, 23], that can drive the recruitment of MDSCs into tumor tissue [24, 25].

In this study we sought to investigate the role of MDSCs in A2B receptor-induced tumor angiogenesis. Our results show that A2B receptor stimulation enhances VEGF release and blood vessel density within tumor tissue in melanoma isografts. These effects are, at least in part, dependent on increased numbers of CD11b+Gr1+ MDSCs in tumor tissue. Pharmacological blockade of A2B receptors reduces tumor angiogenesis and MDSCs accumulation in tumors, leading to a significant delay in the melanoma growth. These effects are associated with improved anti-tumor immune responses and enhanced response to anti-VEGF treatment.

## RESULTS

### A2B receptor regulates tumor infiltration of T cells and suppressive myeloid cells

**Figure 1:**
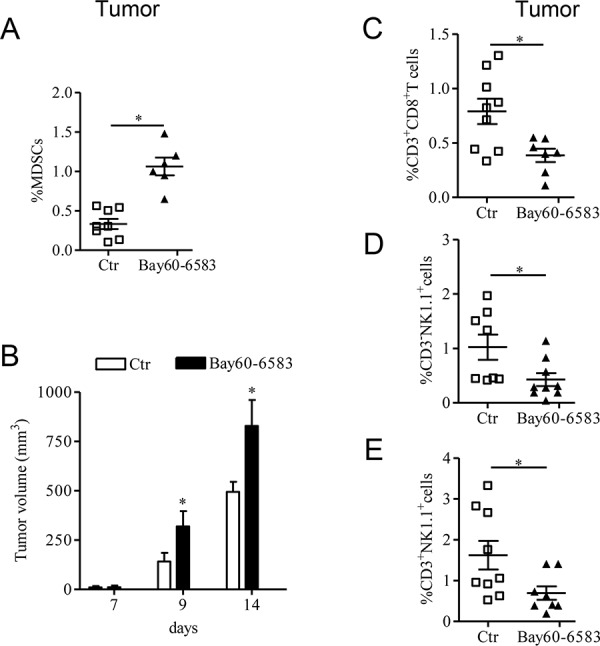
A2B receptor promotes tumor growth by altering the number of tumor-infiltrated immune cells **A.** Percentage of MDSC [CD11b positive (+) Gr1+] cells analyzed by FACS in tumor tissue of C57Bl6j mice bearing melanoma isografts and treated with Bay60-6583 0.2mg/kg or vehicle (ctr) from day 7 after tumor cells injection. **B.** Tumor growth was monitored during the treatment; at the end of the treatment (14 days after tumor cell implantation), mice were sacrificed to collect tumors. **C, D and E.** Percentage of CD3+CD8+ T cells, CD3 negative (−) NK1.1+ cells and CD3+NK1.1+ cells, respectively, analyzed by FACS in the tumor tissues of control and Bay60-6583-treated mice. Data are from two independent experiments and represent mean ± SEM (*n* = 6–9 per group). *p < 0.05.

In tumor-bearing mice as well as in cancer patients, tumor growth correlates with a prominent presence of immunosuppressive cells, including MDSCs, which is accompanied by a decrease of tumor-infiltrating CD8+ T cells [26]. In our previous study we showed that the A2B receptor has a critical role in regulating MDSCs tumor accumulation in mice [22]. In this study, using B16.F10-bearing mice, we confirmed that tumor lesions of mice locally treated with the selective A2B receptor ligand Bay60-6583 [22, 27, 28] (0.2 mg/kg, p.t.) showed increased accumulation of MDSCs (Figure 1A), accompanied by accelerated tumor growth (Figure 1B). These effects were associated with decreased tumor infiltration of CD8+ T cells, NK1.1+ cells and NKT cells (Figure 1C, 1D and 1E, respectively). These results indicate that activation of tumor A2B receptors modulates the presence of tumor-infiltrating immune cells, favoring immunosuppression, and thereby accelerates tumor growth.

### A2B receptor enhances VEGF release within tumor tissue

**Figure 2:**
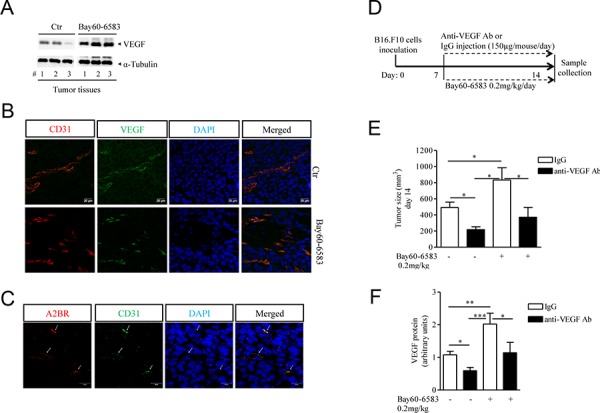
Increased tumor angiogenesis in melanoma-bearing mice treated with Bay60-6583 compared with control mice **A.** Western blotting analysis of VEGF protein expression in melanoma tissue lysates harvested from mice treated with Bay60-6583 0.2 mk/kg or vehicle (ctr). **B.** Immunofluorescence staining of CD31 and VEGF double-positive vessels in melanoma tissue sections of mice treated with Bay60-6583 or vehicle are shown. Magnification 63x. Scale bars represent 20 μm. **C.** Immunofluorescence staining of CD31 and A2B receptor in melanoma sections is shown. Magnification 63x. Scale bars represent 20 μm. **D.** C57Bl6j mice implanted with B16.F10 melanoma cells (day 0), treated from day 7 with Bay60-6583 (0.2 mg/kg p.t.) or vehicle, were injected with anti-VEGF antibody (150 μg/mouse i.p.) or IgG control. At day 14 mice were sacrificed to collect tumor tissues for further analyses. **E.** Tumor volume measured at day 14 after tumor cell implantation of mice treated with Bay60-6583 or vehicle receiving anti-VEGF antibody or IgG as above. **F.** Analysis of VEGF protein expression in melanoma tissue harvested from mice treated as described above. Data are from three independent experiments and represent mean ± SEM (*n* = 6–10 per group). *p < 0.05, **p < 0.01 and ***p < 0.001.

To better characterize the mechanism by which A2B adenosine receptor promotes tumor growth we examined tumor angiogenesis in melanoma-bearing mice treated with Bay60-6583 compared with controls. As shown in Figure 2A, VEGF expression was significantly increased in tumor tissues of mice treated with Bay60-6583 compared to controls. Consistent with enhanced VEGF expression, Bay60-6583-treated mice exhibited higher vessel density than control mice, as assessed by staining melanoma tissue sections for endothelial marker CD31 and VEGF (Figure 2B). Moreover, endothelial cells (CD31+) in tumor sections expressed A2B receptor (Figure 2C). These results support the critical role of the A2B receptor in inducing the release of VEGF from endothelial cells, in line with previous studies [10, 11]. Furthermore, under our experimental conditions, we found that fibroblasts highly populate melanoma lesions (data not shown and unpublished results). Fibroblasts, together with endothelial cells, can also contribute to produce pro-angiogenic factors [29–31, and unpublished results]. Conversely, we were unable to demonstrate VEGF expression in B16.F10 melanoma cells, used in this model.

When VEGF was blocked with a specific antibody in melanoma-bearing mice (Figure 2D) the pro-tumor activity of Bay60-6583 was significantly attenuated (Figure 2E). Treatment of mice with anti-VEGF antibody had significant antitumor activity (Figure 2E), and decreased VEGF levels in tumors of Bay60-6583-treated and vehicle-treated animals (Figure 2F). Taken together, these results indicate that A2B receptor activation stimulates angiogenesis at least in part by inducing VEGF secretion from tumor stromal cells, including endothelial cells and possibly other cell populations.

### MDSCs contribute to the pro-angiogenic effects of Bay60-6583 *in vivo*

**Figure 3:**
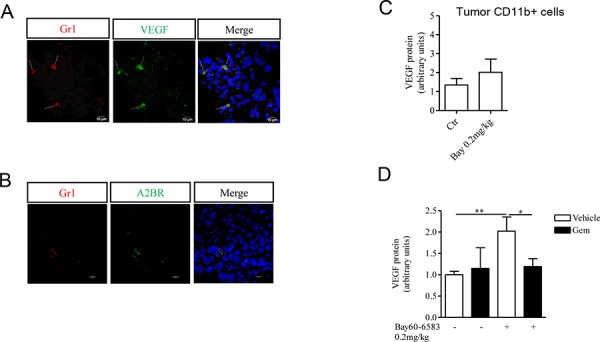
CD11b+Gr1+ cells produce VEGF, that contributes to tumor angiogenesis **A.** Immunofluorescence staining of Gr1+ cells with VEGF in melanoma tissue sections. Magnification 63x. Scale bars represent 10 μm. **B.** Immunofluorescence staining of Gr1+ cells and A2B receptor in melanoma tissue sections. Magnification 63x. Scale bars represent 10 μm. **C.** VEGF protein expression determined by Western blotting in CD11b+ cells isolated from tumor tissues of mice treated with Bay60-6583 or vehicle. **D.** CD11b+Gr1+ cells were depleted by administering gemcitabine (gem, 120mg/kg, i.p.) in melanoma-bearing mice treated with Bay60-6583 or vehicle. Western blotting analysis of VEGF protein expression in melanoma tissue lysates harvested from mice treated as above. Data are from two independent experiments and represent mean ± SEM (*n* = 5–10 per group). *p < 0.05, **p < 0.01.

MDSCs play a pivotal role in inducing tumor angiogenesis by producing pro-angiogenic factors including VEGF [18, 20]. MDSCs expressed VEGF, as demonstrated by co-localization of Gr1 and VEGF staining in melanoma sections (Figure 3A). Since we detected A2B receptor expression in Gr1+ cells (Figure 3B), we evaluated whether Bay60-6583 treatment could directly modulate the release of VEGF from MDSCs. Therefore we analyzed the expression of VEGF in tumor CD11b+ cells isolated from Bay60-6583-treated mice and control mice by Western blotting. While CD11b+ cells did express VEGF, no difference in VEGF expression was found in CD11b+ cells isolated from tumors of Bay60-6583-treated mice compared with those of control mice (Figure 3C).

We previously found that tumor-infiltrating CD11b+Gr1+ cells are increased in Bay60-6583-treated mice [22] (Figure 1A). Therefore, to determine whether the increased number of tumor MDSCs contribute to the enhanced tumor angiogenesis mediated by A2B receptor stimulation, we administered gemcitabine to deplete MDSCs in melanoma-bearing mice treated with Bay60-6583 or vehicle [22, 32–34]. We previously showed that depletion of MDSCs significantly reduces the pro-tumor activity of A2B receptor agonist [22]. Gemcitabine treatment was well tolerated and at the dose we used did not significantly alter the percentage of other cells, such as CD3+, CD4+, CD8+ T and NK1.1+ cells in spleen or the viability of B16.F10 cells [22]. A significant reduction of VEGF expression in tumor tissue was observed in Bay60-6583-treated mice receiving gemcitabine (Figure 3D).

Thus, our results suggest that A2B receptor-mediated stimulation of tumor angiogenesis is, at least in part, due to accumulation of MDSCs within the tumor microenvironment.

### STAT3 activation is enhanced in melanoma tissue of Bay60-6583-treated mice

**Figure 4:**
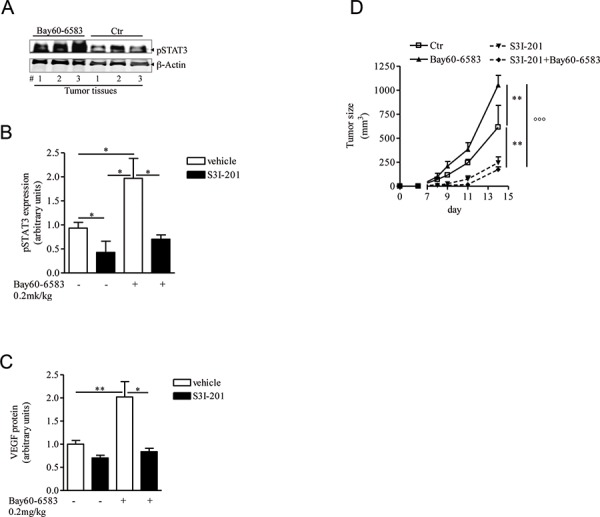
STAT3 activation is enhanced in melanoma tissues of mice treated with Bay60-6583 **A and B.** phospho-STAT3 (pSTAT3) protein expression analysis in melanoma tissues of mice treated with Bay60-6583 or vehicle (ctr), receiving the STAT3 inhibitor S3I-201 5mg/kg i.p. or vehicle. **C.** VEGF protein expression analysis in melanoma tissues of mice treated with Bay60-6583 or vehicle and receiving the STAT3 inhibitor S3I-201. **D.** Melanoma volume was monitored during the treatment with S3I-201 in mice receiving Bay60-6583 or vehicle. Data are from three independent experiments and represent mean ± SEM (*n* = 6–12) *p < 0.05, **p < 0.01 and°°°*p* < 0.001.

In hypoxic tumor microenvironments, several signals can trigger VEGF-A expression and secretion, including among others STAT3 [35]. Tumors in mice treated with Bay60-6583 showed increased phospho-STAT3 (p-STAT3) levels compared with control mice (Figure 4A and 4B). The transcription factor STAT3 translocates into the nucleus where it directly binds to DNA sequences upon phosphorylation at tyrosine 705. STAT3 controls the expression of angiogenic factors, including VEGF, in tumors [36]. We therefore examined the involvement of STAT3 in A2B-mediated effects in our model. Melanoma-bearing mice were treated with highly selective STAT3 inhibitor S3I-201, which preferentially inhibits STAT3 phosphorylation and STAT3 DNA-binding activity [37–39]. S3I-201 suppressed p-STAT3 in melanoma tissue compared to vehicle-treated tumors (Figure 4B). Treatment with S3I-201 significantly inhibited VEGF expression levels in melanoma tissue of Bay60-6583-treated and control mice (Figure 4C). These effects were associated with a marked inhibition of tumor burden in both groups (Figure 4D).

### PSB1115 enhances the efficacy of anti-angiogenic therapy by preventing MDSCs accumulation

**Figure 5:**
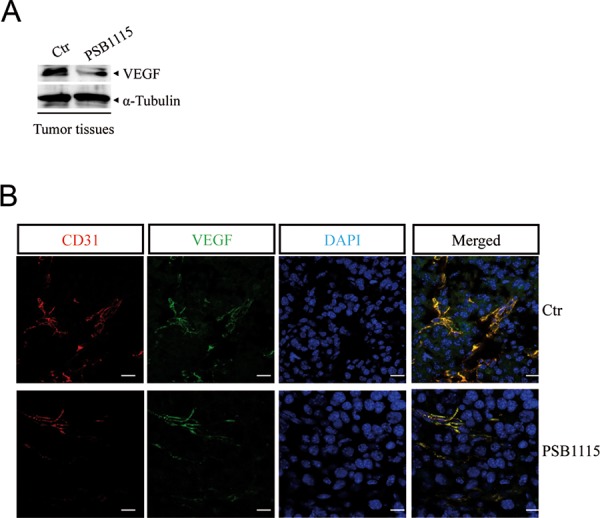
A2B receptor blockade with PSB1115 inhibits tumor angiogenesis **A.** VEGF protein expression in melanoma tissues of mice treated with the A2B receptor antagonist PSB1115 (1 mg/kg) compared with control. **B.** Representative immunofluorescence images of CD31 and VEGF double-positive blood vessels in melanoma sections from control mice or mice treated with PSB1115. Scale bars represent 20 μm

Next, we tested whether pharmacological inhibition of A2B receptors could reduce tumor angiogenesis. Mice bearing B16.F10 tumors were injected with the selective A2B receptor antagonist PSB1115 [22, 40, 41] (1 mg/kg) every day for one week. VEGF expression was reduced in tumor tissue harvested from mice treated with PSB1115 compared with tumors from control mice (Figure 5A). Accordingly, tumor tissue from PSB1115-treated mice showed significantly reduced microvessel density compared with control tumors, as assessed by staining for endothelial marker CD31 and VEGF (Figure 5B).

**Figure 6:**
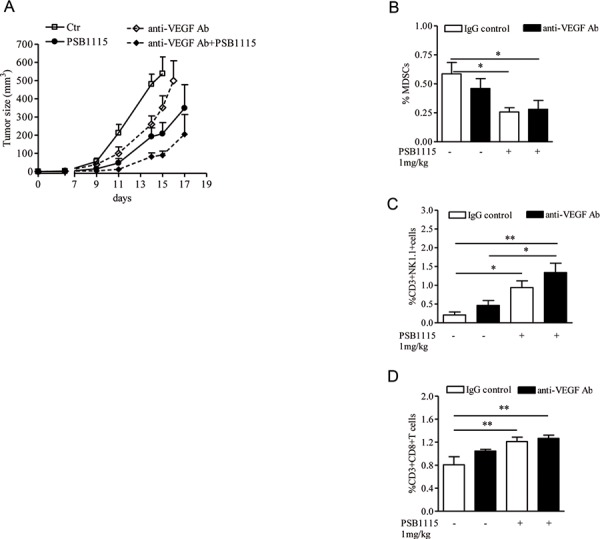
PSB1115 enhances the effects of anti-VEGF treatment by reducing tumor CD11b+Gr1+ cells accumulation **A.** Mice inoculated with B16.F10 melanoma cells were treated with PSB1115 or vehicle in combination with anti-VEGF antibody (150 μg/mouse). Tumor volume was measured at various times during treatment. **B, C and D.** percentage of MDSCs or CD3+NK1.1+ cells or CD3+CD8+ T cells, respectively, analyzed by FACS in melanoma tissues of mice treated as above. Data are from three independent experiments and represent mean ± SEM (*n* = 9–12 per group). *p < 0.05, **p < 0.01.

Although targeting VEGF can suppress angiogenesis and tumor growth, the effectiveness of anti-VEGF therapy is inconsistent and variable among different tumor types [42, 43]. In the clinic, VEGF inhibitors are effective in renal cell carcinoma (RCC) [44] but have limited efficacy in other solid tumors. Accumulation of Gr1+ myeloid cells in the tumor microenvironment increases tumor angiogenesis [18, 20] and mediates tumors refractoriness to anti-VEGF treatment [45, 46]. We previously demonstrated that blocking the A2B receptor can efficiently reduce tumor accumulation of MDSCs and prevent T-cells suppression in mice with melanoma isografts [22]. Thus, we reasoned that PSB1115 may potentiate the effects of anti-VEGF treatment when used in combination with it. Melanoma-bearing mice were treated with PSB1115 (1mg/kg, p.t.), or anti-VEGF antibody (150 μg/mouse, i.p. every two days) or both. PSB1115 alone reduced tumor growth (Figure 6A). Treatment of mice with anti-VEGF antibody delayed tumor growth compared with controls (Figure 6A). The combination of anti-VEGF and PSB1115 inhibited tumor growth more efficiently than anti-VEGF or PSB1115 alone (Figure 6A). Mice treated with both agents showed reduced number of tumor-infiltrating MDSCs (Figure 6B) and increased percentages of CD8+T cells and NKT cells (Figure 6C and 6D). Altogether, these results suggest that targeting A2B receptors can limit tumor growth by reducing tumor angiogenesis and MDSC-mediated immune suppression. Our results suggest that A2B receptor inhibition may potentiate the anti-tumor effects of VEGF inhibitors.

## DISCUSSION

We demonstrate that the A2B receptor plays a pivotal role in inducing tumor angiogenesis, thereby accelerating tumor growth. In addition to promoting the accumulation of MDSCs in tumor microenvironment, A2B stimulation induces VEGF expression *in vivo*. Neutralization of VEGF is effective in limiting the pro-tumor activity of an A2B receptor agonist. Several cell types may contribute to A2B-induced VEGF secretion. Our results indicate that MDSCs, which accumulated in tumor lesions upon A2B receptor stimulation, critically contribute to the enhanced tumor angiogenesis. Depletion of Gr1+CD11b+ cells reduces VEGF expression in tumor tissues, leading to a significant delay in the tumor progression. However, direct stimulation of A2B in CD11b+ cells isolated *ex vivo* did not induce VEGF secretion. There are at least 2 possible explanation for this: 1) the effect of A2B receptor may be limited to inducing accumulation / recruitment of MDSCs, which then produce and secrete VEGF in response to other stimuli; 2) MDSC may require additional factors in addition to A2B stimulation to induce VEGF production (e.g., hypoxia/HIF1-α). One of the main transcriptional activators of VEGF expression in hypoxic tumor lesions is STAT3 [35, 36]. We observed that STAT3 activation is enhanced by A2B receptor stimulation and it is necessary for A2B-induced angiogenesis and VEGF production. Our data do not indicate whether activation of STAT3 is a direct effect of A2B receptor stimulation or is mediated by other factors indirectly induced by A2B. However, pharmacological blockade of A2B receptors inhibited tumor angiogenesis and significantly improved anti-tumor immune surveillance in tumor-bearing mice. This effect is mediated in large part by reduced numbers of MDSCs in tumor microenvironment. A combination regimen of an A2B antagonist and anti-VEGF antibody was more effective than either agent alone in suppressing angiogenesis and tumor growth.

**Figure 7:**
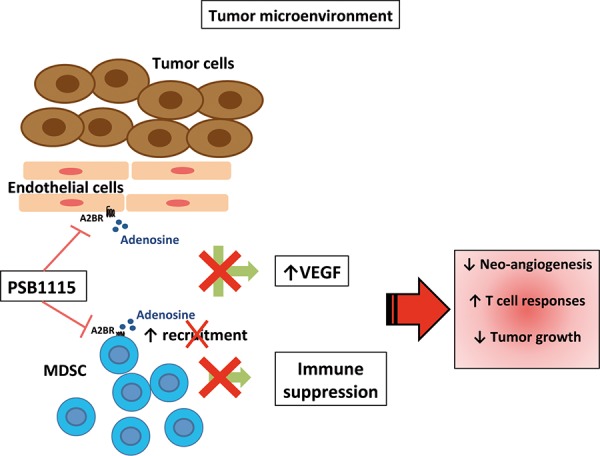
Blockade of A2B receptors with the selective antagonist PSB1115 reduces VEGF release from tumor stromal cells, including endothelial cells. PSB1115 limits the number of tumor MDSCs, which promote immune suppression and contribute to tumor angiogenesis.

The critical role of the A2B receptor in inducing VEGF release in tumor-bearing hosts was demonstrated by Ryzhov and colleagues [13] in A2B deficient mice. Moreover the same authors suggested that host immune cells were crucial in releasing VEGF in an A2B-dependent manner [13]. In line with these studies, we confirmed the important role of A2B receptor in promoting tumor angiogenesis and tumor growth. Importantly, we highlight the critical role of MDSCs in mediating these effects. Myeloid-derived suppressor cells, together with Tregs, are well-known immunoregulatory cells that populate tumor lesions, suppressing protective T-cell responses and promoting tumor growth [16, 17]. It is also documented that MDSCs promote tumor growth by inducing tumor angiogenesis and invasion [18–20]. Generation and accumulation of these cells in tumor-bearing animals and cancer patients is mediated by tumor-derived inflammatory factors and growth factors that accumulate in the tumor environment [24, 26]. MDSCs in turn produce immunosuppressive and pro-angiogenic factors which promote further recruitment of MDSCs into tumor lesions. Hypoxia induces production of adenosine, which directly inhibits T cells functions and induces Tregs in the tumor microenvironment via A2A receptor activation [6, 47]. Recent evidence has emerged that adenosine/adenosine receptors pathways have a critical role in controlling MDSC numbers in tumors. A positive feedback between hypoxia, adenosine and MDSC has been also delineated. MDSCs produce extracellular adenosine [15] that can in turn regulate the expansion/recruitment of additional MDSCs within tumor microenvironment through the A2B receptor [13, 22]. A similar positive feedback exists between hypoxia, VEGF and MDSC [48]. In this study, we show that A2B stimulation promotes intratumoral VEGF production from endothelial cells, which express A2B receptor, while melanoma cells do not. In addition, our data indicate that MDSC, which accumulated into tumor lesions upon A2B stimulation, contribute to release VEGF (Figure 7). We are currently investigating the possible role of tumor-associated fibroblasts, which are abundant in our B16.F10 model tumors. By using a pharmacological approach, we demonstrate that targeting A2B receptors reduces tumor VEGF and limits the number of tumor MDSCs, interrupting the positive feedback circuitry that promotes angiogenesis and MDSC-mediated immune suppression in the tumor environment (Figure 7). These results highlight the critical role of A2B receptor, together with A2A receptor, in contributing to the pro-tumor effects of adenosine. On this point, it should be emphasized that targeting the A2A-A2B receptor axis could be a more general therapeutic approach to overcome adenosine-induced immune-suppression and angiogenesis in tumors.

Mechanistically, we observed that STAT3 activation is enhanced in A2B stimulated mouse tumors. Inhibition of STAT3 in melanoma-bearing mice prevents the effects of A2B receptor stimulation on VEGF levels. STAT3 activation is implicated in tumor progression and angiogenesis [35, 36]. It is well documented that STAT3 upregulates the expression of inflammatory and pro-angiogenic genes, including VEGF-A [35, 36]. On the other hand, pro-angiogenic factors are themselves activators of STAT3. It is unclear whether A2B stimulation can directly signal through STAT3 in our model. Published results indicate that adenosine increases IL-10-induced STAT3 activation and signaling in M2-like macrophages in an A2B receptor-dependent manner [49]. A2B receptor stimulation enhances IL-10 production in tumor tissue [22]. It is possible that A2B stimulation could enhance IL-10-mediated STAT3 activation in at last some cell types in our tumor model.

Anti-VEGF agents can be effective in controlling tumor progression in human cancer patients [44, 50–52]. However, the efficacy of these therapies can be limited by various mechanisms, including accumulation of MDSCs in tumor lesions [45]. In this work we demonstrate that pharmacological blockade of A2B receptor inhibits tumor growth in mice, by simultaneously reducing tumor angiogenesis and improving anti-tumor immune surveillance. These effects are associated with decreased number of CD11b+Gr1+ cells in tumors. In accordance with the role of MDSC in promoting tumor angiogenesis, reduction of these cells in the tumor tissue upon A2B receptor blockade improves the therapeutic efficacy of anti-VEGF treatment. Since VEGF itself recruits MDSC to tumors [21, 24], reduced VEGF expression may contribute to the inhibition of MDSC recruitment caused by A2B receptor antagonists.

Taken together, our results reveal that A2B receptor-induced accumulation of Gr1+ myeloid cells in tumors is responsible not only for MDSC-mediated immune suppression in the tumor microenvironment but also for enhanced tumor angiogenesis. Our findings support the therapeutic potential of A2B antagonists, in combination with immunotherapy and/or anti-angiogenic agents.

## MATERIALS AND METHODS

### Animals and cells

C57Bl6j mice (6–8 week-old females) were obtained from Charles River (Charles River, Lecco, Italy). B16-F10 melanoma cells from American Type Culture Collection (LGC Standards srl., Milan, Italy) were injected s.c. into the flank of mice. Tumor volume was calculated using the formula 4/3π × (long diameter / 2) × (short diameter / 2) [53]. Experiments using animals were approved by Italian Health Ministry and conducted according to institutional animal care guidelines, Italian Law 26/2014 based on the European Community Law for Animal Care 2010/63/UE. Bay60-6583 (0.2 mg/kg) [22, 27, 28] or PSB1115 (1 mg/kg) [22, 40, 41] (both from Sigma Aldrich, Milan, Italy) were injected p.t. on day 7 after melanoma cells injection; phosphate-buffered saline (PBS) containing DMSO was used as control for Bay60-6583. For MDSCs depletion, 120 mg/kg gemcitabine (Sigma Aldrich) [22, 32–34] was administered i.p. on day 7 and every three days until endpoint. For VEGF neutralization experiments, 150 μg of anti-VEGF A antibody (BioLegend, Campoverde, Milan, Italy) was administered i.p. every day starting from day 7. IgG was used as control. To block STAT3 signalling S3I-201 (5 mg/kg) (Sigma Aldrich) [37–39] was administered i.p. on day 7 and every two days until endpoint; phosphate-buffered saline containing DMSO was used as control.

### Flow cytometry

Single cell suspensions were prepared from harvested melanoma tissues. Tissues were dissected and digested with collagenase 1U/ml, passed through 70-μm cell strainers and red blood cells (RBC) were lysed. Cell samples were pre-incubated with anti-mouse CD16/CD32 (eBioscience, San Diego, CA, USA) to block non-specific Fc-mediated interactions. Antibodies against CD11c-FITC, CD11b-PeCy5.5, Gr1-PE or Gr1-allophycocyanin, CD3-PeCy5.5; CD8-allophycocyanin or CD8-PE; CD4-allophycocyanin; NK1.1-PE were obtained from eBioscience and BioLegend. Data were acquired with a FACSCalibur flow cytometer (BD Biosciences).

### Isolation of cells

Melanoma tissues were collected aseptically and digested with collagenase 1U/ml. Cells were passed through 70 μm filters, followed by RBC lysis. CD11b+MDSCs were isolated by positive magnetic selection of CD11b+ cells (CD11b EasySep Isolation kit; Stem Cell Tech). Purity of CD11b+ cells was checked by flow cytometry and was routinely around 90%.

### Western blot analysis

Tumor tissues were homogenized in RIPA buffer (RIA Precipitation Buffer), centrifuged and protein concentrations in supernatants were determined by Bio-Rad protein assay. Forty micrograms of total proteins were fractionated through 10% denaturing polyacrylamide gels and then transferred electrophoretically to nitrocellulose membranes (Immobilon-NC, Millipore). Anti-VEGF (A-20) (Santa Cruz Biotechnology, DBA, Milan, Italy) or anti-tubulin (or anti-actin) (Sigma-Aldrich) primary antibodies were used (Santa Cruz Biotechnology). VEGF expression was also measured in the total cell lysate from isolated CD11b+ cells. Immunoreactive protein bands were visualized by enhanced chemiluminescence reagents (Amersham Pharmacia Biotech, Buckinghamshire, UK) and analyzed to Las4000 (GE Healthcare Life Sciences).

### Immunofluorescence analysis by confocal microscopy

Tumor tissue were fixed with 0.5% neutral buffered paraformaldehyde at 4°C for 2 hours and then incubated with 15% sucrose at 4°C overnight. Samples were then frozen in Optimum Cutting Temperature (OCT) medium (Pella, Milan, Italy) and 10–20 μm thick sections were cut. For nonspecific binding sites, slides were blocked in PBS / 20% normal goat serum (GIBCO BRL) or 5% Bovine Serum Albumin (BSA) containing 0.5% TritonX-100 for 30 minutes at room temperature. Sections were stained overnight with primary antibodies: VEGF (A-20) (1:100; Santa Cruz Biotechnology) and CD31 (MEC 7.46) (1:200; Abcam, Cambridge, UK) or Gr1 (Ly-6G, clone RB6–8C5) (1:100; eBioscience) at 4°C in a humidified chamber and detected with Alexa Fluor^®^ 488 Goat Anti-Rabbit IgG (H+L) (1:1000) secondary antibodies and Alexa Fluor^®^ 555 Goat Anti-Rat IgG (H+L) (1:1000) (Life Technologies, Italy), respectively, for 2 hours at room temperature. In some experiments, FITC anti-mouse CD31 (MEC 13.3) (1.100, BioLegend) was used. Primary antibody for A2B receptor: A2B-R (N-19) (1:50; Santa Cruz Biotechnology) was detected with secondary antibodies DyeLight™ 549-conjugated AffinePure Donkey anti-goat IgG (H+L) or DyeLight™ 488-conjugated AffinePure Donkey anti-goat IgG (H+L) (Jackson Immunoresearch Laboratories Inc.). DAPI was used to counterstain nuclei. In all staining experiments, isotype-matched IgG and omission of the primary Ab was used as controls. Slides were observed using a Zeiss LSM 710 Laser Scanning Microscope (Carl Zeiss MicroImaging GmbH). Samples were vertically scanned from the bottom of the coverslip with a 63 × (1.40 NA) Plan-Apochromat oil-immersion objective. Images were generated with Zeiss ZEN Confocal Software (Carl Zeiss MicroImaging GmbH).

### Statistical analysis

Data are from at least two-three independent experiments and results are expressed as mean ± SEM. Two-tailed Student’s *t* test (2-group comparisons) or ANOVA (> 2-group comparisons) were performed as appropriate. *P* values < 0.05 were considered significant.
